# A retrospective analysis of the safety of tacrolimus use and its optimal cut-off concentration during pregnancy in women with systemic lupus erythematosus: study from two Japanese tertiary referral centers

**DOI:** 10.1186/s13075-023-03256-8

**Published:** 2024-01-04

**Authors:** Takehiro Nakai, Nanase Honda, Eri Soga, Sho Fukui, Ayako Kitada, Naoto Yokogawa, Masato Okada

**Affiliations:** 1 Immuno-Rheumatology Center, St. Luke’s International Hospital, 9-1 Akashi-Cho, Chuo-Ku, Tokyo, Japan; 2 Department of Rheumatic Diseases, Tokyo Metropolitan Tama Medical Center, Tokyo, Japan; 3 Department of Obstetrics and Gynecology, Tokyo Metropolitan Tama Medical Center, Tokyo, Japan; 4 Department of General Medicine, Kyorin University School of Medicine, Tokyo, Japan; 5 Division of Rheumatology, Inflammation, and Immunity, Department of Medicine, Brigham and Women’s Hospital and Harvard Medical School, Boston, MA USA; 6 Department of Rheumatology, Institute of Medicine, University of Tsukuba, Ibaraki, Japan

**Keywords:** Systemic lupus erythematosus, Lupus nephritis, Pregnancy outcome, Tacrolimus

## Abstract

**Background:**

Tacrolimus is one of the major treatment options for systemic lupus erythematosus (SLE) and is considered to be a pregnancy-compatible medication. Since little is known about tacrolimus safety during pregnancy complicated by SLE, this study was designed.

**Methods:**

We included SLE pregnant patients who were followed up at two Japanese tertiary referral centers. We performed multivariate logistic regression analysis to assess each adverse pregnancy outcome (APO) risk. Moreover, we assessed the influence of tacrolimus on the APO ratio in pregnant patients with lupus nephritis, and the impact of combined tacrolimus-aspirin therapy on the APO ratio relative to patients exclusively administered tacrolimus.

**Results:**

Of the 124 pregnancies, 29 were exposed to tacrolimus. Multivariate analysis showed no statistical difference in APO ratio. (overall APO: adjusted odds ratio [aOR], 0.69; 95% confidence interval [CI], 0.23–2.03; *p* = 0.50; maternal APO: aOR, 1.17; 95% CI, 0.36–3.83; *p* = 0.80; neonatal APO: aOR, 1.10; 95% CI, 0.38–3.21; *p* = 0.86; PROMISSE APO: aOR, 0.50; 95% CI, 0.14–1.74; *p* = 0.27).

Blood pressure and estimated glomerular filtration rate (eGFR) during pregnancy and after delivery did not differ between the two groups. Receiver operating characteristic (ROC) curve showed that tacrolimus concentration > 2.6 ng/ml was related to reduced preterm birth rate. (AUC = 0.85, 95% CI: 0.61–1.00, sensitivity: 93% and specificity: 75%).

Regarding effect of tacrolimus on lupus nephritis during pregnancy, tacrolimus showed no increased risk of APO, blood pressure or eGFR during pregnancy and after delivery. (overall APO: OR, 1.00; 95% CI, 0.25–4.08; *p* = 0.98; maternal APO: OR 1.60, 95% CI, 0.39–6.64; *p* = 0.51; neonatal APO: OR, 0.71; 95% CI, 0.17–3.03; *p* = 0.65, PROMISSE APO: OR, 0.50; 95% CI, 0.08–3.22; *p* = 0.47).

Tacrolimus-aspirin combination therapy showed a protective tendency against hypertensive disorders during pregnancy, preeclampsia and low birth weight.

**Conclusions:**

Tacrolimus use during pregnancy with SLE and lupus nephritis showed no significant influence on APO, blood pressure, or renal function; therefore tacrolimus may be suitable for controlling lupus activity during pregnancy. In addition, when using tacrolimus during pregnancy, we should aim its trough concentration ≥ 2.6 ng/ml while paying careful attention to possible maternal side effects of tacrolimus.

**Trial registration:**

Retrospectively registered.

**Supplementary Information:**

The online version contains supplementary material available at 10.1186/s13075-023-03256-8.

## Introduction

Systemic lupus erythematosus (SLE) is a systemic disease that affects multiple organs, including the renal and neurological systems [1].

Although pregnancies in patients with SLE tend to have poorer outcomes than those in healthy individuals, advancements in the management of SLE during pregnancy have led to improved outcomes [2].

Reproductive guidelines published by the European League Against Rheumatism (EULAR), the American College of Rheumatology (ACR), and the British Society for Rheumatology all indicate that prednisolone, hydroxychloroquine, azathioprine, cyclosporin, and tacrolimus are safe to use during pregnancy [3–5].

Tacrolimus inhibits T-cell activation by reducing calcineurin activity and subsequent transcription of gene encoding interleukin-2. Given the crucial roles of not only B cells but also T cells, specifically granzyme K + CD8 T cells in the pathogenesis of SLE [6, 7], tacrolimus was proven to be effective and safe to use as an induction and maintenance therapy [8–10] This has led to its widespread application as a main treatment option for SLE [1].

While all three previously mentioned reproductive guidelines advocate for the safety of tacrolimus during pregnancy [3–5], these advisories were established based on the safety profile of tacrolimus observed in organ transplant recipients, suggesting favorable pregnancy outcomes (preeclampsia: 15.1%, live birth rate: 68%, spontaneous abortion, 12%, stillbirth/perinatal death: 3%) However, the incidence of adverse pregnancy outcomes (APOs) varied due to transplant-associated comorbidities and concomitant immunosuppression. Notably, many of these reports lack a comprehensive comparison group that did not use tacrolimus [11–13] Nevertheless, the specific implications of tacrolimus usage during pregnancy in the context of SLE and lupus nephritis remain to be clarified, with even the most substantial study including only 25 SLE pregnancies with tacrolimus exposure [14].

Furthermore, calcineurin inhibitors including tacrolimus are occasionally implicated in triggering endothelial damage owing to oxidative stress, inhibited production of endothelial nitric oxide, and endothelial inflammation elicited by toll-like receptor 4 signaling [15–17]. Since endothelial damage was proven to be related to placental vascular abnormalities [18] tacrolimus use during pregnancy could potentially be associated with augmented prevalence of APOs. Aspirin is reported to prevent endothelial damage [19]; however, no studies have addressed the effect of tacrolimus-aspirin combination therapy on APO risk reduction.

In addition, tacrolimus use can sometimes affect the control of hypertension, glucose tolerance, and renal function [20, 21], but in pregnant patient with SLE and lupus nephritis, sufficient data is lacking to elucidate the effects of tacrolimus use on blood pressure, glucose homeostasis, and renal function during gestation. To prevent tacrolimus-related side effects, it is imperative to monitor tacrolimus trough concentration [22]. However, no studies to date have explicitly investigated the significance of monitoring this concentration in pregnant individuals with SLE to prevent APOs. Regarding neonatal care, tacrolimus was reported to cross the placenta and be potentially related to adverse neonatal pregnancy outcomes [23].

To address this knowledge gap, our group previously explored tacrolimus usage in lupus complicated pregnancies as a single-center study [24]. However, the findings of that study were limited by the number of pregnancies included. This limitation could have restricted the analysis of tacrolimus use during pregnancy with lupus based on less common APOs. These included PROMISSE (Pregnancy Outcome: Biomarkers in Antiphospholipid Antibody Syndrome and Systemic Lupus Erythematosus) APO; correlation between tacrolimus trough concentration and each APO, and evaluation of group differences in the APO ratio between patients with lupus nephritis and those without, as well as those treated with a tacrolimus-aspirin combination therapy.

Therefore, to further encourage clinicians to consider tacrolimus in pregnancies complicated by SLE, we undertook a multi-center study with the following four objectives: assess the safety of tacrolimus in pregnancies associated with SLE, evaluate the safety of tacrolimus in pregnancies complicated by lupus nephritis, determine the optimal cutoff value for tacrolimus trough concentration during pregnancy, and examine the impact of combined tacrolimus and aspirin therapy on the prevalence of APO.

## Methods

### Study design

We conducted a retrospective analysis using the complete health records of patients with SLE who were treated at the Tokyo Metropolitan Tama Medical Center (Tokyo, Japan) and St. Luke’s International Hospital (Tokyo, Japan) between April 2010 and September 2022. Patients who received perinatal SLE interventions and obstetrical management at each centers, and subsequently delivered at the same institutions, were included in this study. We excluded patients who declined enrollment in the study or lacked data on pregnancy outcomes. All data were extracted from the electronic medical records of the Tokyo Metropolitan Tama Medical Center (Tokyo, Japan) and St. Luke’s International Hospital (Tokyo, Japan).

All the authors were involved in data collection. TN and NH took charge of data collection and reconfirmed the accuracy of the data.

We divided the patients according to the tacrolimus exposure during pregnancy and evaluated the effects of tacrolimus on the prevalence of APOs in patients representing all SLE populations and those with lupus nephritis. We also assessed the impact of combined tacrolimus-aspirin therapy on the APO ratio relative to those exclusively administered tacrolimus.

We further employed receiver operating characteristic (ROC) curve analysis to ascertain the optimal concentration of tacrolimus during pregnancy.

The study was approved by the Ethics Committee of St. Luke’s International Hospital. Written informed consent was obtained from all participants (approval No. 22-R077).

### SLE diagnosis

We used all three major classification criteria: the 1997 ACR, Systemic Lupus International Collaborating Clinics 2012, and 2019 EULAR)/ACR because diagnosis of SLE based on single classification criteria could lead to misdiagnosis of true SLE patient [25–28].

### Tacrolimus prescription

Tacrolimus was administered in accordance with the Japanese treatment guidelines, which specifies a standard dose of 3 mg once daily for patients with SLE. However, the treating rheumatologist may lower this dosage in case of concerns about potential side effects of tacrolimus and if the tacrolimus trough concentration exceeds 10 ng/ml.

### Data collected

We collected data on demographics; duration between SLE onset and conception; organ manifestation (mucocutaneous, joint and muscular, renal, serositis, neurological, and hematological); and immunological profiles. Additionally, we collected data on maternal pregnancy outcomes (lupus flare; hypertensive disorders of pregnancy; gestational diabetes mellitus; preeclampsia; hemolysis, elevated liver enzymes and low platelets (HELLP) syndrome; and maternal death). Data on maternal renal function, maternal blood pressure, and neonatal pregnancy outcomes (spontaneous abortion, missed abortion, stillbirth, preterm birth, low birth weight, small for gestational age (SGA), Apgar score < 7 at 1 or 5 min, and major malformation) were also collected. All pregnancy data were monitored from six months preconception to six months postpartum.

### Definition of the term

We collected data on four types of APO, namely overall, maternal, neonatal, and PROMISSE APO. Maternal APO was defined as occurrence of at least one of the following: SLE flare during pregnancy or until 6 months after delivery (BILAG category A in at least one organ system), hypertensive disorders of pregnancy, gestational diabetes mellitus, preeclampsia, HELLP syndrome, or maternal death during pregnancy.

The diagnosis of hypertensive disorders of pregnancy, gestational diabetes mellitus, preeclampsia, HELLP syndrome were based on the American College of Obstetricians and Gynecologists (ACOG) practice guideline [29, 30].

We defined neonatal APO as neonates with at least one of the following: preterm birth (live birth before 37 weeks of gestation), spontaneous abortion (death of the fetus at < 22 weeks of gestation), missed abortion (in utero death of the embryo or fetus with retained conception products at < 22 weeks of gestation), still birth (death of the fetus at ≥ 22 weeks of gestation), low birth weight (birth weight < 2500 g), SGA (body weight and/or height below the 10th percentile for gestational age), Apgar score less than 7 at 1 or 5 min, or major malformation (serious anomaly of surgical or cosmetic importance [31]). Overall APO was defined as any of the maternal and/or neonatal APOs.

PROMISSE APO was also assessed to identify the severe form of neonatal APO. PROMISSE APO was defined as neonates with any of the following: fetal death after 12 weeks of gestation, neonatal death before hospital discharge, preterm delivery, or termination of pregnancy < 36 weeks due to hypertensive disorder during pregnancy/preeclampsia/placental problem or SGA [32].

### Definition of SLE remission

Zen et al.’s definitions of remission in SLE was used to assess remission of SLE [33].

### Statistical analysis

We divided the patients into two groups according to tacrolimus use. Categorical data are presented as numbers and percentages, whereas continuous data are presented as median values and interquartile ranges. To compare categorical variables, both Fisher's exact test and the Chi-square test were employed. To compare continuous variables, the Mann–Whitney U test was utilized. We used logistic regression models for calculating the odds ratio (OR) for each APO according to tacrolimus use during pregnancy. Furthermore, we performed the multivariate analysis, using variables previously identified as being linked with an increased APO ratio. Specifically, these variables encompass the presence of renal manifestation, highest dosage of prednisolone administered during pregnancy, and employment of hydroxychloroquine throughout the gestational period [34–36].

ROC curves were used for identifying cutoff values for preventing each APO in pregnant patients treated with tacrolimus. Using ROC curves, we aimed to determine the optimal cutoff value of tacrolimus trough concentration during pregnancy that yielded the best sensitivity and specificity.

Furthermore, we used propensity score matching for reducing the effects of confounding variables between the two groups in a 1:2 ratio using calipers of width equal to 0.2 of the standard deviation of the logit of the propensity score. We calculated the propensity score based on the covariables with differences between the two groups that were thought to be related to the prevalence of APOs: presence of renal manifestation, maximum prednisolone dosage during pregnancy, and hydroxychloroquine usage during pregnancy [34–36].

ORs for the prevalence of each APO according to tacrolimus use during pregnancy were calculated using logistic regression analysis.

Furthermore, Fisher’s exact test and the Chi-square test were used to compare differences in categorical variables, while the Mann–Whitney U test was used to compare differences in continuous variables among pregnant patients with lupus nephritis based on tacrolimus exposure. Similarly, Fisher’s exact test and the Chi-square test were used to compare differences in categorical variables, and the Mann–Whitney U test was used to compare differences in continuous variables among pregnant patients with SLE on tacrolimus, based on the concomitant use of aspirin. All statistical analyses were performed using EZR (version 2.7–1; Saitama Medical Center, Jichi Medical University, Saitama, Japan)—a graphical user interface for R (The R Foundation for Statistical Computing, Vienna, Austria). Statistical significance was set at *p* < 0.05.

## Results

### Population characteristics

We included 124 pregnancies in 97 pregnant patients. Twenty-nine pregnancies received tacrolimus during pregnancy. (Supplementary Fig. S1) There were no remarkable differences in the median age at conception, body mass index (BMI), or duration of SLE. However, the pregnant patients on tacrolimus were more likely to experience lupus nephritis and lupus flare at conception compared with those without exposure (tacrolimus exposure versus no exposure; renal manifestation: 51.7% versus 17.9%, *p* < 0.01; lupus nephritis class III/IV: 20.7% versus 6.3%, *p* = 0.053; and lupus flare at conception: 19.2% versus 1.4%, *p* < 0.01).

There were no major difference in most of the immunological profiles except for positivity for anti-dsDNA antibody and anti-La/SSB antibody (anti-dsDNA antibody: 82.8% versus 64.2%, *p* = 0.098, anti-La/SSB antibody: 0.0% versus 21.7%, *p* = 0.032) (Table 1).

**Table 1:** Baseline characteristics

	tacrolimus exposure		
Factor	(-)	( +)	*p* value
Number of patients	95	29	
***Epidemiological findings***			
Age at conception, years	33.0 [29.1, 35.0]	33.0 [31.0, 36.0]	0.17
BMI	19.8 [18.6, 21.3]	20.4 [18.1, 23.4]	0.52
Duration of SLE (days)	2614 [1514, 4985]	2548 [1003, 4277]	0.58
Smoking history (%)	10 (10.5)	2 (6.9)	0.73
Previous spontaneous abortion (%)	16 (16.8)	8 (27.6)	0.31
Previous anti-hypertensive medication use (%)	1 (1.1)	5 (17.2)	< 0.01
Multiparous (%)	34 (36.6)	11 (37.9)	1.00
Infertility treatment (%)	22 (23.2)	8 (27.6)	0.81
Any flare at conception (%)	1 (1.4)	5 (19.2)	< 0.01
Remission at conception (%)	46 (65.7)	13 (50.0)	0.24
***Organ manifestation***			
Joint/muscular manifestation (%)	59 (62.1)	20 (69.0)	0.65
Skin/mucocutaneous manifestation (%)	70 (73.7)	17 (58.6)	0.19
Renal manifestation (%)	17 (17.9)	15 (51.7)	< 0.01
Lupus nephritis class III/IV (%)	6 (6.3)	6 (20.7)	0.053
Serositis (%)	17 (17.9)	8 (27.6)	0.38
Neurological manifestation (%)	8 (8.4)	2 (6.9)	1.00
Hematological manifestation (%)	79 (83.2)	21 (72.4)	0.31
***Immunological findings***			
Anti-dsDNA Ab (%)	61 (64.2)	24 (82.8)	0.098
Anti-RNP Ab (%)	22 (36.7)	8 (42.1)	0.888
Anti-Sm Ab (%)	28 (31.5)	8 (29.6)	1.00
Anti-Ro/SSA Ab (%)	55 (59.1)	21 (72.4)	0.29
Anti-La/SSB Ab (%)	13 (21.7)	0 (0.0)	0.032
LAC (%)	9 (9.8)	4 (13.8)	0.51
Anti-CL Ab (%)	21 (23.1)	6 (21.4)	1.00
Anti-CLβ2GPI Ab (%)	13 (14.0)	2 (6.9)	0.52
Low C3 (%)	55 (59.1)	18 (62.1)	0.94
Low C4 (%)	69 (74.2)	22 (75.9)	1.00

*BMI* body mass index, *CL* cardiolipin, *LAC* lupus anticoagulant, *SLE* systemic lupus erythematosus

### Treatment regimen

As presented in Supplementary Table S1, glucocorticoid dosage in the first trimester was higher in the pregnant on tacrolimus group compared with the unexposed group. Additionally, more patient on tacrolimus used hydroxychloroquine in the first trimester.

(glucocorticoid dosage [prednisolone equivalent], 5.00 (5.00–10.00) mg/day versus 5.00 (1.00–7.75) mg/day, *p* = 0.025, hydroxychloroquine: 69.0% versus 28.4%, *p* < 0.01) (Supplementary Table S1).

There were no differences in the usage of other immunosuppressants at conception between the two groups.

Within the cohort exposed to tacrolimus, tacrolimus was administered post-conception to two patients to manage lupus flares during pregnancy.

(one during the second trimester and the other during the third trimester).

Of note, one patient with tacrolimus exposure used mizoribine and another one without tacrolimus exposure used mycophenolate mofetil at conception. Both of them conceived unintentionally and underwent iatrogenic abortion after a careful discussion on the possible malformation risk. Except for the two, all patients were treated with pregnancy-compatible medications.

In addition, three pregnancies were exposed to belimumab at conception but were discontinued once pregnancy was confirmed because there were scarce data to confirm that belimumab was safe to continue throughout pregnancy [3–5, 37–40]. Additionally, we have proved that belimumab discontinuation could be possible [41].

### Prevalence of APO

The prevalence of overall APO showed no statistical difference between the two groups (overall APO: 55.2% versus 48.4%, *p* = 0.67). Maternal APO was more frequently observed in the pregnancies with tacrolimus exposure compared with those without (44.8% versus 24.2%, *p* = 0.056), possibly due to the increased SLE flare rate during pregnancy in patients treated with tacrolimus (27.6% versus 8.4%, *p* = 0.017). No remarkable difference between the two groups was noted in the prevalence of hypertensive disorders of pregnancy, preeclampsia, HELLP syndrome, and oligohydramnios (hypertensive disorders of pregnancy: 24.1% versus 13.7%, *p* = 0.29; preeclampsia: 10.3% versus 5.3%, *p* = 0.39; HELLP syndrome: 0.0% versus 2.1%, *p* = 1.00; and oligohydramnios: 3.4% versus 8.4%, *p* = 0.68). No maternal death was observed in either group (Table 2).

**Table 2:** Prevalence of adverse pregnancy outcome according to tacrolimus exposure

	Tacrolimus exposure			Logistic regression (univariate)			Logistic regression (multivariate)		
Factor	(-)	( +)	*p* value	OR	95% CI	*P* value	aOR	95% CI	*P* value
n	95	29							
Overall APO (%)	46 (48.4)	16 (55.2)	0.67	1.31	0.57–3.02	0.525	0.69	0.23–2.03	0.50
Maternal APO (%)	23 (24.2)	13 (44.8)	0.056	2.54	1.07–6.07	0.035	1.17	0.36–3.83	0.80
Neonatal APO (%)	42 (44.2)	15 (51.7)	0.62	1.35	0.59–3.11	0.48	1.10	0.38–3.21	0.86
PROMISSE APO (%)	24 (25.3)	5 (17.2)	0.52	0.62	0.21–1.79	0.38	0.50	0.14–1.74	0.27
Flare during pregnancy (%)	8 (8.4)	8 (27.6)	0.017	4.14	1.39–12.3	0.010			
Flare after delivery (%)	3 (3.4)	2 (8.7)	0.27	2.73	0.43–17.4	0.29			
Hypertensive disorder during pregnancy (%)	13 (13.7)	7 (24.1)	0.29	2.01	0.72–5.64	0.19			
Preeclampsia (%)	5 (5.3)	3 (10.3)	0.39	2.08	0.47–9.27	0.34			
HELLP syndrome (%)	2 (2.1)	0 (0.0)	1.00	NA	NA	NA			
Gestational DM (%)	8 (8.4)	6 (20.7)	0.14	2.84	0.90–9.0	0.076			
Oligohydramnios (%)	8 (8.4)	1 (3.4)	0.68	0.38	0.05–3.21	0.38			
Maternal death (%)	0 (0.0)	0 (0.0)	NA	NA	NA	NA			
Total duration of gestation (days)	267.0 [257.5, 275.0]	266.0 [213.0, 273.0]	0.17	NA	NA	NA			
Live birth (%)	88 (92.6)	23 (79.3)	0.088	0.31	0.09–0.10	0.049			
Preterm birth (%)	16 (17.8)	5 (20.0)	1.00	1.16	0.38–3.54	0.80			
Spontaneous abortion (%)	1 (1.1)	2 (7.4)	0.12	7.52	0.66–86.3	0.11			
Missed abortion (%)	3 (3.2)	1 (3.7)	1.00	1.18	0.12–11.8	0.89			
Iatrogenic abortion (%)	4 (4.2)	3 (10.3)	0.35	2.63	0.55–12.5	0.22			
Still birth (%)	0 (0.0)	0 (0.0)	NA	NA	NA	NA			
Planned C section (%)	14 (15.7)	3 (13.0)	1.00	0.80	0.21–3.07	0.75			
Emergency C section (%)	28 (31.5)	13 (56.5)	0.048	2.83	1.11–7.24	0.030			
Weight at birth (g)	2709 [2411, 3016]	2812 [2165, 2943]	1.00	NA	NA	NA			
Low birth weight (%)	31 (35.2)	8 (34.8)	1.0	0.91	0.37–2.57	0.97	0.67	0.19–2.33	0.53
SGA (%)	15 (17.0)	4 (17.4)	0.37	1.02	0.31–3.45	NA			
Apgar score 1 m	8.0 [8.0, 8.0]	8.00 [8.0, 8.0]	0.29	NA	NA	NA			
Apgar score 5 m	9.0 [9.0, 9.0]	9.00 [9.0, 9.0]	0.92	NA	NA	NA			
Major malformation (%)	1 (1.1)	1 (4.3)	0.37	3.95	0.24–65.8	0.34			
Death of neonate (%)	0 (0.0)	1 (4.3)	0.21	NA	NA	NA			

*aOR* adjusted odds ratio, *APO* adverse pregnancy outcome, *DM* diabetes mellitus, *HELLP* hemolysis, elevated liver enzymes and low platelets, *NA* not available, *OR* odds ratio, *PROMISSE* Predictors of Pregnancy Outcome: Biomarkers in Antiphospholipid Antibody Syndrome and Systemic Lupus Erythematosus, *SGA* small for gestational age

The prevalence of neonatal APO, including PROMISSE APO, did not differ between the two groups (neonatal APO: 51.7% versus 44.2%, *p* = 0.62; PROMISSE APO: 17.2% versus 25.3%, *p* = 0.52). The live birth rate was slightly lower in the pregnant patients on tacrolimus compared with those without exposure (79.3% versus 92.6%, *p* = 0.088) possibly due to a higher iatrogenic abortion ratio (10.3% versus 4.2%, *p* = 0.35). However, no major difference was observed in the prevalence of preterm birth, spontaneous abortion and missed abortion (preterm birth: 20% versus 17.8%, *p* = 1.00; spontaneous abortion: 7.4% versus 1.1%, *p* = 0.12; and missed abortion: 3.7% versus 3.2%, *p* = 1.00).

Multivariate analysis, adjusted for renal manifestations, the highest dosage of prednisolone administered during pregnancy, and the use of hydroxychloroquine throughout the gestational period, showed no statistical difference in overall, maternal, neonatal, and PROMISSE APOs, even with exposure to tacrolimus during pregnancy (overall APO: adjusted OR (aOR), 0.69; 95% confidence interval [CI], 0.23–2.03; *p* = 0.50; maternal APO: aOR, 1.17; 95% CI, 0.36–3.83; *p* = 0.80;neonatal APO: aOR, 1.10; 95% CI, 0.38–3.21; *p* = 0.86; PROMISSE APO: aOR, 0.50; 95% CI, 0.14–1.74; *p* = 0.27). In addition, tacrolimus exposure showed no statistical increase in low birth weight baby (aOR, 0.67; 95% CI, 0.19–2.33; *p* = 0.53).

Even after propensity score matching, the OR of each APO according to tacrolimus use showed no statistical increase in the prevalence of each APO (overall APO: OR, 1.00; 95% CI, 0.31–3.21; *p* = 1.00; maternal APO: OR, 1.16; 95% CI, 0.32–4.21; *p* = 0.82; neonatal APO: OR, 2.04; 95% CI, 0.63–6.66; *p* = 0.24; PROMISSE APO: OR, 1.00; 95% CI, 0.22–4.61; *p* = 1.00; hypertensive disorders of pregnancy: OR, 1.24; 95% CI, 0.26–5.96; *p* = 0.79; preeclampsia: OR, 1.00; 95% CI, 0.08–11.9; *p* = 1.00) (Supplementary Tables S2, S3, S4).

### Change in blood pressure and renal function during pregnancy

**Fig. 1:**
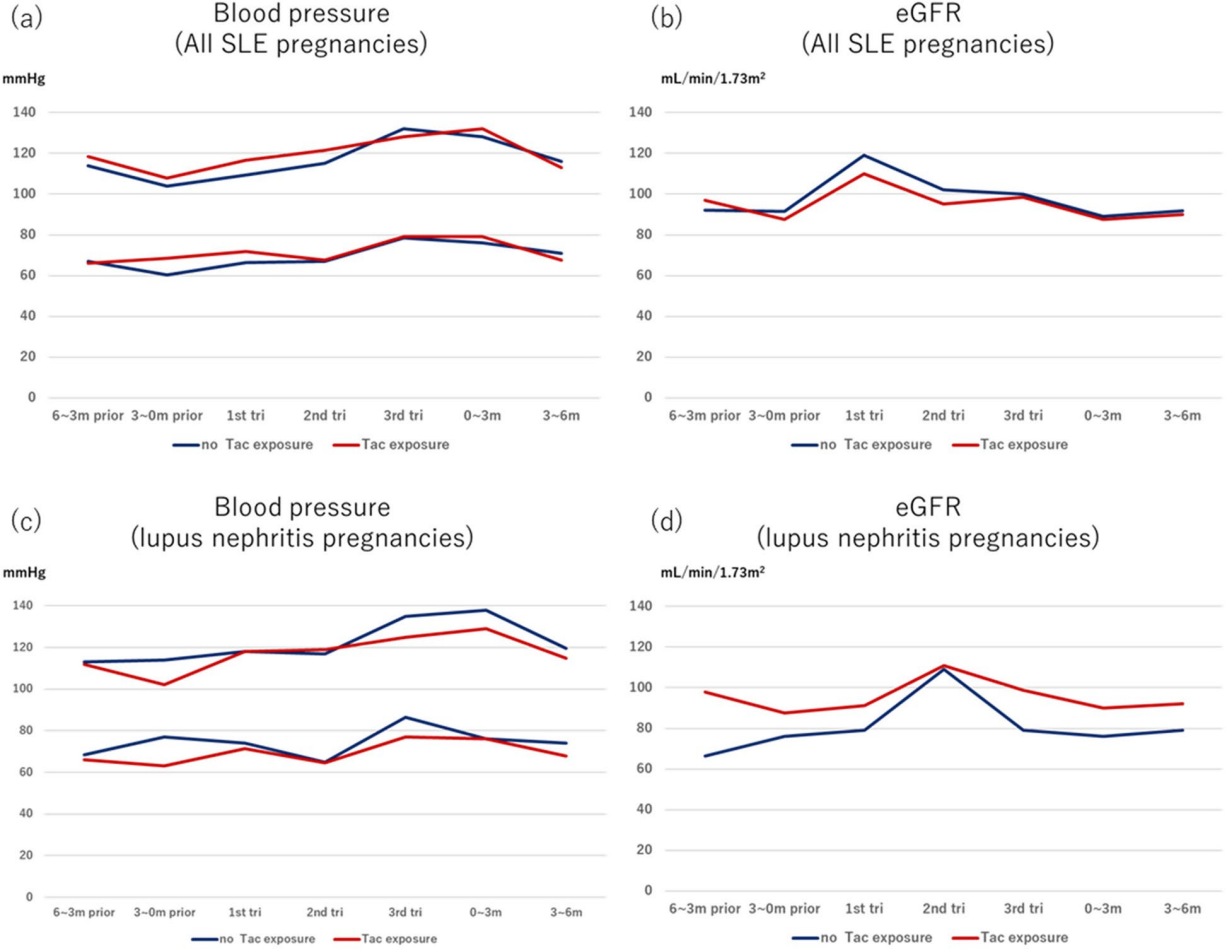
Change in the blood pressure before and during pregnancy and after delivery. **a** Change in blood pressure of all SLE pregnancy. **b** Change in eGFR of all SLE pregnancy. **c** Change in blood pressure of lupus nephritis pregnancy. **d** Change in eGFR of lupus nephritis pregnancy. eGFR: estimated glomerular filtration rate, SLE: systemic lupus erythematosus

No major differences were observed in blood pressure and estimated glomerular filtration rate (eGFR) value between the two groups at each time point of pregnancy and after delivery (Fig. 1a, b).

### Tacrolimus trough concentration and APO ratio

Using ROC curves, we aimed to identify the appropriate cutoff value for maximum tacrolimus trough concentrations during pregnancy to reduce each APO in the patients exposed to tacrolimus.

**Fig. 2:**
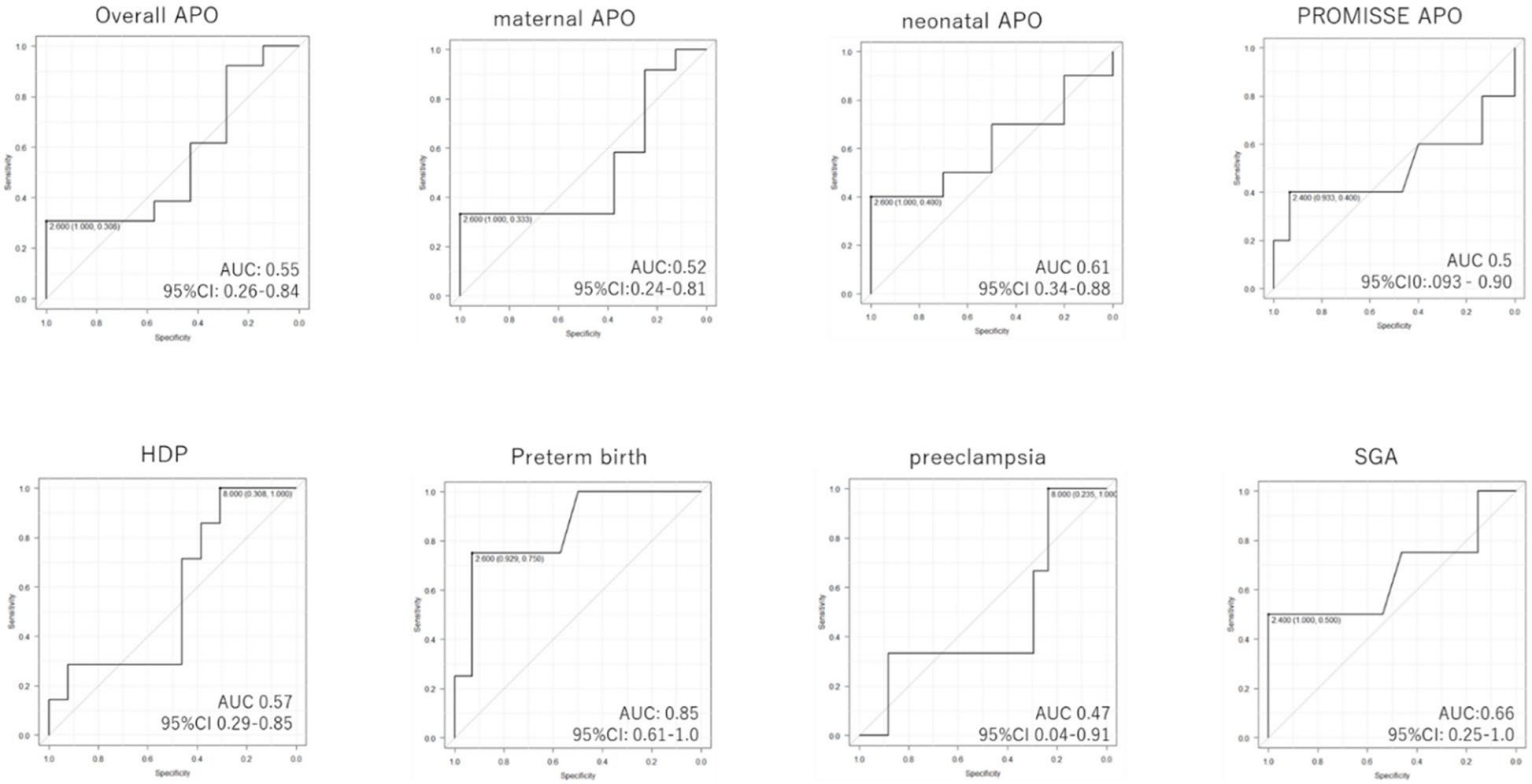
ROC curve for the maximum tacrolimus trough concentration during pregnancy and each adverse pregnancy outcome. APO: adverse pregnancy outcome, AUC: are under the curve. CI: confidence interval HDP: hypertensive disorders during pregnancy. ROC: receiver operating characteristic, SGA: small for gestational age

As shown in Fig. 2, most APOs including hypertensive disorder during pregnancy and preeclampsia exhibited no significant association with the peak tacrolimus trough concentration during gestation.

SGA showed a relatively high area under the curve (AUC, 0.66, 95% CI: 0.25–1.00), albeit lower than 0.7.

Preterm birth exhibited high AUC (AUC = 0.85, 95% CI: 0.61–1.00). Moreover, the optimal cutoff value for tacrolimus trough concentration to prevent preterm birth was ≥ 2.6 ng/mL (sensitivity: 93% and specificity: 75%) (Fig. 2).

Moreover, we employed the Mann–Whitney U test among pregnant patients exposed to tacrolimus to re-evaluate the association between tacrolimus trough concentration and preterm delivery, which showed that maximum tacrolimus trough concentration during pregnancy was lower in the pregnant patients with preterm birth compared with those without (the pregnant patients with preterm birth 2.50 [2.02, 3.20] ng/mL versus those without preterm birth 5.10 [3.38, 7.97] ng/mL, *p* = 0.03).

### Prevalence of APO according to tacrolimus exposure in patients with lupus nephritis

We also analyzed the prevalence of APO according to tacrolimus use in patients with lupus nephritis.

Of the 32 pregnancies with lupus nephritis, 15 were exposed to tacrolimus during pregnancy. As shown in Supplementary Table S5, approximately 40% of the pregnant patients in each group experienced lupus nephritis class III/IV (patients with lupus nephritis on tacrolimus versus those without tacrolimus exposure: 40.0% versus 35.3%, *p* = 1.00). In addition, there were no major differences in epidemiological findings, organ manifestation, or immunological profiles except for anti-Ro/SSA antibody positivity (patients with lupus nephritis on tacrolimus versus those without tacrolimus exposure: 80.0% versus 31.2%, *p* = 0.011). Although hydroxychloroquine was more frequently used in the pregnant patients with lupus nephritis exposed to tacrolimus (patients with lupus nephritis on tacrolimus versus those without tacrolimus exposure: 73.3% versus 29.4%, *p* = 0.034) (Supplementary Table S6), no major difference in APO or changes in blood pressure/eGFR during pregnancy and after delivery were noted between the two groups (overall APO: 53.3% versus 52.9%, *p* = 1.00; maternal APO: 46.7% versus 35.3%, *p* = 0.77; neonatal APO: 33.3% versus 41.2%, *p* = 0.93; PROMISSE APO: 13.3% versus 23.5%, *p* = 0.66; hypertensive disorders of pregnancy: 20.0% versus 23.5%, *p* = 1.00; preeclampsia: 13.3% versus 17.6%, *p* = 1.00) (Table 3). (Fig. 1c, d).

**Table 3:** Prevalence of adverse pregnancy outcome in patients with lupus nephritis according to the tacrolimus exposure

	Tacrolimus exposure in pregnant with lupus nephritis			Logistic regression model		
Factor	(-)	( +)	*P* value	OR	95% CI	*P* value
N	17	15				
Overall APO (%)	9 (52.9)	8 (53.3)	1.00	1.00	0.25–4.08	0.98
Maternal APO (%)	6 (35.3)	7 (46.7)	0.77	1.60	0.39–6.64	0.51
Neonatal APO (%)	7 (41.2)	5 (33.3)	0.93	0.71	0.17–3.03	0.65
PROMISSE.APO (%)	4 (23.5)	2 (13.3)	0.66	0.50	0.08–3.22	0.47
Flare during pregnancy (%)	3 (17.6)	2 (13.3)	1.00	0.72	0.10–5.01	0.74
Flare after conception (%)	1 (6.2)	2 (15.4)	0.57	2.73	0.22–34	0.44
Hypertensive Disorders of Pregnancy (%)	4 (23.5)	3 (20.0)	1.00	0.81	0.15–4.4	0.81
Preeclampsia (%)	3 (17.6)	2 (13.3)	1.00	0.72	0.10–5.01	0.74
Eclampsia (%)	1 (5.9)	0 (0.0)	1.00	NA	NA	NA
HELLP syndrome (%)	0 (0.0)	0 (0.0)	NA	NA	NA	NA
Gestational DM (%)	2 (11.8)	3 (20.0)	0.65	1.87	0.27–13	0.53
Oligohydramnios (%)	1 (5.9)	1 (6.7)	1.00	1.14	0.07–20	0.93
PROM (%)	0 (0.0)	1 (6.7)	0.47	NA	NA	NA
Maternal death (%)	0 (0.0)	0 (0.0)	NA	NA	NA	NA
Live birth	16 (94.1)	13 (86.7)	0.91	0.41	0.03–5	0.48
Total duration of gestation (days)	264.0 [259.0, 268.0]	271.0 [262.0, 276.0]	0.045	NA	NA	NA
Preterm birth (%)	3 (18.8)	1 (7.1)	0.60	0.33	0.31–3.64	0.37
Spontaneous abortion (%)	0 (0.0)	0 ( 0.0)	NA	NA	NA	NA
Missed abortion (%)	0 (0.0)	1 (6.7)	0.47	NA	NA	NA
Iatrogenic abortion (%)	1 (5.9)	1 (6.7)	1.00	1.14	0.07–20.0	0.93
Still birth (%)	0 (0.0)	0 (0.0)	NA	NA	NA	NA
Planned C section (%)	5 (31.2)	2 (15.4)	0.41	0.40	0.06–2.52	0.33
Emergency C section (%)	4 (25.0)	8 (61.5)	0.07	4.80	0.98–23.5	0.05
Height at birth (cm)	46.9 [45.2, 47.4]	49.5 [46.5, 50.5]	0.10	NA	NA	NA
Weight at birth (g)	2627.5 [2438.0, 2770.3]	2914.0 [2812.0, 3025.0]	0.03	NA	NA	NA
Low birth weight (%)	6 (37.5)	2 (15.4)	0.24	0.30	0.05–1.86	0.20
SGA (%)	1 (6.2)	2 (15.4)	0.57	2.73	0.22–34	0.44
Apgar score (1 min)	8.0 [8.0, 8.0]	8.0 [8.0, 8.0]	0.77	NA	NA	NA
Apgar score (5 min)	9.0 [9.0, 9.0]	9.0 [9.0, 9.0]	0.88	NA	NA	NA
Major malformation (%)	0 (0.0)	1 (7.7)	0.45	NA	NA	NA
Death of neonate (%)	0 (0.0)	0 (0.0)	NA	NA	NA	NA

*APO* adverse pregnancy outcome, *CI* confidence interval, *DM* diabetes mellitus, *NA* not available, *HELLP* hemolysis, elevated liver enzymes and low platelets*, OR* odds ratio, *PROMISSE* Predictors of Pregnancy Outcome: Biomarkers in Antiphospholipid Antibody Syndrome and Systemic Lupus Erythematosus, *PROM* Premature rupture of membranes, *PS* propensity score, *SGA* small for gestational age

The logistic regression model also showed no increased prevalence of each APO according to the tacrolimus exposure.

(overall APO: OR, 1.00; 95% CI, 0.25–4.08; *p* = 0.98; maternal APO: OR 1.60, 95% CI, 0.39–6.64; *p* = 0.51; neonatal APO: OR, 0.71; 95% CI, 0.17–3.03; *p* = 0.65, PROMISSE APO: OR, 0.50; 95% CI, 0.08–3.22; *p* = 0.47).

### Tacrolimus + aspirin combination therapy

Of the 29 pregnancies on tacrolimus, 16 were concomitantly treated with aspirin.

Compared with the tacrolimus-only group, the pregnant patients on tacrolimus + aspirin combination therapy tended to have a lower frequency of experiencing hypertensive disorders of pregnancy and preeclampsia, but no statistical difference was noted possibly due to the limited number of patients included (tacrolimus + aspirin versus tacrolimus only: hypertensive disorders of pregnancy, 18.8% versus 30.8%, *p* = 0.67; preeclampsia. 6.2% versus 15.4%, *p* = 0.57).

In addition, the birth weight of neonates born from patients receiving tacrolimus + aspirin tended to be higher compared with those from mothers receiving tacrolimus only (2882 [2532, 3025] g versus 2577 [2123, 2889] g, *p* = 0.17).

Logistic regression model analysis further demonstrated that the combination therapy of tacrolimus and aspirin appeared to reduce the incidence of hypertensive disorders of pregnancy, preeclampsia, and low birth weight infants when compared with the group treated solely with tacrolimus, but no statistical difference was noted possibly due to the small sample size. (hypertensive disorders of pregnancy: OR, 0.52; 95% CI, 0.93–2.90; *p* = 0.46; preeclampsia: OR, 0.37; 95% CI, 0.03–4.57; *p* = 0.44; low birth weight: OR, 0.30; 95% CI, 0.05–1.80; *p* = 0.19) (Table 4).

**Table 4:** Tacrolimus-aspirin combination therapy and its effect on adverse pregnancy outcomes

	Tacrolimus + aspirin combination therapy			logistic regression analysis		
Factor	Tacrolimus only	Tacrolimus + aspirin	*p* value	OR	95% CI	*p* value
n	13	16				
Overall APO (%)	7 (53.8)	9 (56.2)	1.00	1.10	0.25–4.8	0.90
Maternal APO (%)	5 (38.5)	8 (50.0)	0.80	1.60	0.36–7.07	0.54
Neonatal APO (%)	8 (61.5)	7 (43.8)	0.56	0.49	0.11–2.16	0.34
PROMISSE APO (%)	2 (15.4)	3 (18.8)	1.00	1.27	0.18–9.02	0.81
Flare during pregnancy (%)	3 (23.1)	5 (31.2)	0.70	1.52	0.29–8.03	0.63
flare after delivery (%)	1 (10.0)	1 (7.7)	1.0	0.75	0.04–13.7	0.85
Hypertensive Disorders of Pregnancy (%)	4 (30.8)	3 (18.8)	0.67	0.52	0.93–2.90	0.46
Preeclampsia (%)	2 (15.4)	1 (6.2)	0.57	0.37	0.03–4.57	0.44
Eclampsia (%)	0 (0.0)	0 (0.0)	NA	NA	NA	NA
HELLP syndrome (%)	0 (0.0)	0 (0.0)	1.00	NA	NA	NA
Gestational DM (%)	2 (15.4)	4 (25.0)	0.66	1.83	0.28–12.1	0.53
Oligohydramnios (%)	1 (7.7)	0 (0.0)	0.45	NA	NA	NA
Maternal death (%)	0 (0.0)	0 (0.0)	NA	NA	NA	NA
Total duration of gestation (days)	263 [166, 273]	266 [245.8, 272]	0.71	NA	NA	NA
Preterm birth (%)	3 (27.3)	2 (14.3)	0.62	0.44	0.06–3.29	0.43
Spontaneous abortion (%)	1 (8.3)	1 (6.7)	1.00	0.79	0.04–14	0.87
Missed abortion (%)	1 (7.7)	0 (0.0)	0.48	NA	NA	NA
Iatrogenic abortion (%)	1 (7.7)	2 (12.5)	1.00	1.70	0.14–21.3	0.68
Still birth (%)	0 (0.0)	0 (0.0)	NA	NA	NA	NA
Planned C section (%)	1 (10.0)	2 (15.4)	1.00	1.64	0.13–21.1	0.71
Emergency C section (%)	6 (60.0)	7 (53.8)	1.00	0.78	0.15–4.13	0.77
Height at birth (cm)	46.3 [43.8, 49.9]	48.0 [44.0, 50.0]	0.64	NA	NA	NA
Weight at birth (g)	2577 [2123, 2889]	2882 [2532, 3025]	0.17	NA	NA	NA
Low birth weight (%)	5 (50.0)	3 (23.1)	0.22	0.30	0.05–1.80	0.19
SGA (%)	2 (20.0)	2 (15.4)	1.00	0.73	0.08–6.31	0.77
Apgar score > 7 (1 min)	8 (80.0)	13 (100.0)	0.35	NA	NA	NA
Apgar score > 7 (5 min)	9 (90.0)	13 (100.0)	0.89	NA	NA	NA
Major malformation (%)	0 (0.0)	1 (7.7)	1.00	NA	NA	NA
Death of the neonate (%)	1 (10.0)	0 (0.0)	0.44	NA	NA	NA

*APO* adverse pregnancy outcome, *DM* diabetes mellitus, *HELLP* hemolysis, elevated liver enzymes and low platelets*, NA* not available, *OR* odds ratio, *PROMISSE* Predictors of Pregnancy Outcome: Biomarkers in Antiphospholipid Antibody Syndrome and Systemic Lupus Erythematosus, *PROM* Premature rupture of membranes, *SGA* small for gestational age

## Discussion

In this largest to date database regarding tacrolimus use during SLE pregnancy, we have found that tacrolimus usage bore no relation to any deterioration in maternal blood pressure or renal function. In addition, tacrolimus use during SLE pregnancy did not amplify the prevalence of APOs including hypertensive disorders during pregnancy, preeclampsia, or PROMISSE APO in patients with SLE or those with lupus nephritis. Previous reports have indicated the prevalence of each APO in pregnant patients with SLE to be as follows: SLE flare during pregnancy (21.4%–64%), hypertensive disorders of pregnancy (0.99%–45%), gestational diabetes mellitus (0%–11%), preeclampsia (5.4%–20.2%), HELLP syndrome (0.3%–0.66%), preterm birth (9%–56%), spontaneous abortion (0.4%–25%), SGA (10%–28.5%), an Apgar score less than 7 at 1 min (1%–18%) [42], and PROMISSE APO (approximately 19%) [32]. Accordingly, our results align with and are not inferior to these historical accounts of SLE associated pregnancies.

Although tacrolimus usage can occasionally be associated with nephrotoxicity, [20, 43] existing data on its effects during pregnancy remain sparse. One study examining patients with lupus nephritis administered tacrolimus during pregnancy revealed that tacrolimus did not exacerbate eGFR. Nevertheless, the results require cautious interpretation given that the study failed to compare renal function between patients exposed to tacrolimus during pregnancy and those who were not [43]. Our analysis showed no alterations in renal function amongst pregnant lupus patients treated both with and without tacrolimus, revealing no discernible difference in eGFR during pregnancy or post-delivery. Thus, tacrolimus usage appears to pose no risk for renal function.

Measuring tacrolimus trough concentration is important in preventing side effects such as nephrotoxicity [22]. Our data further demonstrated the importance of measuring tacrolimus concentration because we have proven that low tacrolimus trough concentration during pregnancy was related to increased preterm birth risk. Since preterm birth is related to increased risk of chronic kidney disease and vascular disease in the newborn by inhibiting normal organogenesis and vascular tree growth [44, 45], preventing preterm birth is of importance. Furthermore, we evaluated the prevalence of APOs in patients on tacrolimus + aspirin combination therapy and those administered tacrolimus alone. This analysis showed a tendency of tacrolimus + aspirin combination therapy for preventing hypertensive disorders of pregnancy, preeclampsia, and low birth weight neonates, without showing statistical difference possibly due to the small sample size.

Our findings suggest that tacrolimus is not only safe for pregnant women who have undergone organ transplantation but also for those with SLE and lupus nephritis. Furthermore, we demonstrated that maintaining a tacrolimus concentration above 2.6 ng/ml was associated with a decreased ratio of preterm births.

Owing to advancements in the care of pregnancy-associated rheumatic diseases, women with SLE, previously regarded excessively severe to conceive, can now consider motherhood [42]. Given that tacrolimus may present a critical therapeutic strategy for patients with moderately to severely active SLE [46], this result could further encourage social remission of patients with SLE by helping them fulfill their aspirations for becoming mothers.

### Limitations

Our study has several limitations.

First, although we conducted this study as a multi-center study in Japan, the number of patients included was relatively small. Therefore, these results warrant further evaluation in a larger multiethnic cohort.

Based on historical records concerning comparing the effect of hydroxychloroquine and aspirin combination therapy in patient with SLE, hypertensive disorder of pregnancy was observed in 2.2% for those on hydroxychloroquine and aspirin therapy, in contrast to 17.8% for those on hydroxychloroquine only group [47]. Utilizing this data for sample size determination with an alpha level of 0.05 and a power of 0.8, a sample size of *n* = 70 for each group is necessitated and for those with less common APOs, further sample size was necessitated.

Second, we excluded patients who delivered at clinics or other hospitals other than our centers. Since both of our centers are tertiary teaching hospitals that admit patients with relatively severe conditions, the severity of pregnancy tended to be higher and could have influenced APO prevalence.

Third, clinical manifestations of patients on tacrolimus were more severe compared with those unexposed.

Although we have re-analyzed this data using a multivariate logistic regression model and propensity score matching, potential confounding variables might have been inadvertently overlooked.

Finally, a relatively small population was treated with hydroxychloroquine and aspirin probably because of the delayed introduction of hydroxychloroquine in Japan post-October 2015, with 50 of the 124 deliveries occurring prior to the approval of hydroxychloroquine in the country. In addition, more than half of the pregnancies (63/124) gave birth before the publication of the study on the effect of aspirin in preventing preeclampsia (Aug 2017) [48].This result might have affected the APO ratio in our population.

## Conclusion

Tacrolimus exposure during pregnancy did not increase any APO ratio and had no negative impact on blood pressure/renal function during pregnancy and after delivery in pregnant with SLE and lupus nephritis. Therefore, its use might be acceptable in pregnant patients with clinically active lupus. In addition, when using tacrolimus during pregnancy, we should aim its trough concentration ≥ 2.6 ng/ml while paying careful attention to possible maternal side effects of tacrolimus.

### Supplementary Information

**Additional file 1: Supplementary Figure S1.** Patient flowchart. **Supplementary Table S1.** Treatment regimen at each time point of pregnancy. **Supplementary Table S2.** Baseline characteristics before and after propensity score matching. **Supplementary Table S3.** Treatment regimen in the first trimester before and after propensity score matching. **Supplementary Table S4.** Prevalence of adverse pregnancy outcome after propensity score matching. **Supplementary Table S5.** Baseline characteristics of patient with lupus nephritis. **Supplementary Table S6.** treatment regimen in the first trimester in patient with lupus nephritis.

## Data Availability

All data generated/analyzed during this study are included in this article and supplementary data.

## Abbreviations

ACR: American College of Rheumatology
aOR: Adjusted odds ratio
APO: Adverse pregnancy outcome
BMI: Body mass index
CI: Confidence interval
eGFR: Estimated glomerular filtration rate
EULAR: European League against Rheumatism
HELLP: Hemolysis, elevated liver enzymes and low platelets
OR: Odds ratio
PROMISSE: Pregnancy Outcome: Biomarkers in Antiphospholipid Antibody Syndrome and Systemic Lupus Erythematosus
ROC: Receiver operating characteristic
SMD: Standardized mean difference
SGA: Small for gestational age
SLE: Systemic lupus erythematosus
