# Supplementary material for: A retrospective analysis of the safety of tacrolimus use and its optimal cut-off concentration during pregnancy in women with systemic lupus erythematosus: study from two Japanese tertiary referral centers
Source: Arthritis Res Ther. 2024 Jan 4;26:15. doi: 10.1186/s13075-023-03256-8 (PMC10765865; doi:10.1186/s13075-023-03256-8)
Supplement: Supplementary file 1 — Additional file 1: Supplementary Figure S1. Patient flowchart. Supplementary Table S1. Treatment regimen at each time point of pregnancy. Supplementary Table S2. Baseline characteristics before and after propensity score matching. Supplementary Table S3. Treatment regimen in the first trimester before and after propensity score matching. Supplementary Table S4. Prevalence of adverse pregnancy outcome after propensity score matching. Supplementary Table S5. Baseline characteristics of patient with lupus nephritis. Supplementary Table S6. treatment regimen in the first trimester in patient with lupus nephritis. [file 13075_2023_3256_MOESM1_ESM.docx]

**Supplementary Figure S1: patient flowchart**

**
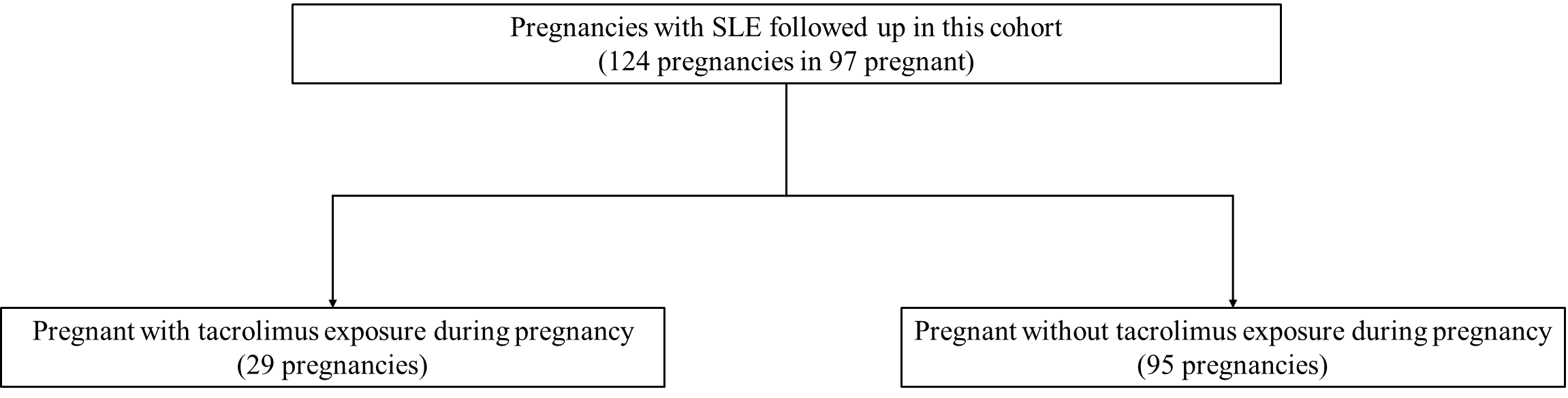
**

**Supplementary Table S1: Treatment regimen at each time point of pregnancy**

|  | 6~3m prior | | | 3~0m prior | | | First trimester | | | second trimester | | | Third trimester | | | 0-3m | | | 3-6m | | |
| --- | --- | --- | --- | --- | --- | --- | --- | --- | --- | --- | --- | --- | --- | --- | --- | --- | --- | --- | --- | --- | --- |
|  | Tacrolimus exposure | | | Tacrolimus exposure | | | Tacrolimus exposure | | | Tacrolimus exposure | | | Tacrolimus exposure | | | Tacrolimus exposure | | | Tacrolimus exposure | | |
| Factor | (-) | (+) | p value | (-) | (+) | p value | (-) | (+) | p value | (-) | (+) | p value | (-) | (+) | p value | (-) | (+) | p value | (-) | (+) | p value |
| n | 61 | 24 |  | 61 | 24 |  | 95 | 29 |  | 86 | 26 |  | 88 | 22 |  | 86 | 24 |  | 76 | 24 |  |
| PSL (mg/day) | 5.00 [1.50, 9.00] | 5.00 [5.00, 6.00] | 0.32 | 5.00 [1.00, 8.50] | 5.00 [4.00, 9.00] | 0.075 | 5.00 [1.00, 7.75] | 5.00 [5.00, 10.00] | 0.025 | 5.00 [1.00, 7.88] | 7.00 [5.00, 11.88] | <0.01 | 5.00 [1.38, 10.0] | 6.00 [5.00, 10.00] | 0.074 | 5.00 [1.50, 10.00] | 8.00 [5.00, 11.25] | 0.064 | 5.00 [1.88, 10.00] | 6.00 [5.00, 9.00] | 0.21 |
| HCQ (%) | 22 (23.9) | 18 (62.1) | <0.01 | 27 (29.3) | 17 (58.6) | <0.01 | 27 (28.4) | 20 (69.0) | <0.01 | 29 (31.9) | 19 (73.1) | <0.01 | 28 (31.5) | 17 (77.3) | <0.01 | 31 (34.8) | 16 (66.7) | 0.01 | 30 (35.7) | 16 (66.7) | 0.013 |
| Tac (%) | 1 (1.6) | 24 (100.0) | <0.01 | 1 (1.6) | 24 (100.0) | <0.01 | 0 (0.0) | 27 (93.1) | <0.01 | 0 (0.0) | 25 (96.2) | <0.01 | 0 (0.0) | 20 (90.9) | <0.01 | 1 (1.2) | 24 (100.0) | <0.01 | 2 (2.7) | 24 (100.0) | <0.01 |
| CyA (%) | 2 (3.3) | 0 (0.0) | 1.00 | 2 (3.3) | 0 (0.0) | 1.00 | 2 (2.1) | 0 (0.0) | 1.00 | 2 (2.3) | 0 (0.0) | 1.0 | 2 (2.3) | 0 (0.0) | 1.00 | 2 (2.3) | 0 (0.0) | 1.00 | 2 (2.7) | 0 (0.0) | 1.00 |
| AZA (%) | 8 (13.1) | 0 (0.0) | 0.10 | 7 (11.5) | 0 (0.0) | 0.18 | 6 (6.3) | 0 (0.0) | 0.33 | 5 (5.8) | 0 (0.0) | 0.59 | 5 (5.7) | 0 (0.0) | 0.58 | 4 (4.7) | 0 (0.0) | 0.58 | 4 (5.3) | 0 (0.0) | 0.57 |
| MMF (%) | 0 (0.0) | 2 (8.3) | 0.077 | 0 (0.0) | 0 (0.0) | NA | 1 (1.1) | 0 (0.0) | 1.0 | 0 (0.0) | 0 (0.0) | NA | 0 (0.0) | 0 (0.0) | NA | 0 (0.0) | 0 (0.0) | NA | 0 (0.0) | 0 (0.0) | NA |
| MZR (%) | 0 (0.0) | 0 (0.0) | NA | 0 (0.0) | 1 (4.2) | 0.28 | 0 (0.0) | 1 (3.4) | 0.23 | 0 (0.0) | 0 (0.0) | NA | 0 (0.0) | 0 (0.0) | NA | 1 (1.2) | 0 (0.0) | 1.00 | 1 (1.3) | 0 (0.0) | 1.00 |
| BEL (%) | 2 (3.3) | 2 (8.3) | 0.32 | 1 (1.6) | 2 (8.3) | 0.19 | 1 (1.1) | 2 (6.9) | 0.14 | 0 (0.0) | 0 (0.0) | NA | 0 (0.0) | 0 (0.0) | NA | 0 (0.0) | 1 (4.2) | 0.22 | 0 (0.0) | 1 (4.2) | 0.24 |
| MTX/ /CY/RTX/plasma exchange (%) | 0 (0.0) | 0 (0.0) | NA | 0 (0.0) | 0 (0.0) | NA | 0 (0.0) | 0 (0.0) | NA | 0 (0.0) | 0 (0.0) | NA | 0 (0.0) | 0 (0.0) | NA | 0 (0.0) | 0 (0.0) | NA | 0 (0.0) | 0 (0.0) | NA |
| IVIg (%) | 0 (0.0) | 0 (0.0) | NA | 0 (0.0) | 0 (0.0) | NA | 0 (0.0) | 0 (0.0) | NA | 0 (0.0) | 0 (0.0) | NA | 0 (0.0) | 1 (4.5) | 0.20 | 0 (0.0) | 1 (4.2) | 0.22 | 0 (0.0) | 0 (0.0) | NA |
| Aspirin (%) | 8 (13.1) | 2 (8.3) | 0.72 | 8 (13.1) | 4 (16.7) | 0.73 | 32 (33.7) | 12 (41.4) | 0.51 | 40 (46.5) | 13 (52.0) | 0.66 | 27 (29.9) | 10 (45.5) | 0.37 |  |  |  |  |  |  |

AZA: azathioprine, BEL: belimumab, CY: cyclophosphamide, CyA: cyclosporine , GC: glucocorticoid, HCQ: hydroxychloroquine, IVIg: intravenous immunoglobulin, MMF: mycophenolate mofetil, MTX: methotrexate, MZR: mizoribine, NA: not available, PE: plasma exchange, PSL: prednisolone, RTX: rituximab, Tac: tacrolimus

**Supplementary Table S2:** Baseline characteristics before and after propensity score matching

|  | tacrolimus exposure (before matching) | | | tacrolimus exposure (after matching) | | |
| --- | --- | --- | --- | --- | --- | --- |
| Factor | (-) | (+) | SMD | (-) | (+) | SMD |
| N | 95 | 29 |  | 34 | 17 |  |
| ***Epidemiological findings*** |  |  |  |  |  |  |
| Age at conception (yo) | 33.0 [29.1, 35.0] | 33.0 [31.0, 36.0] | 0.33 | 33.0 [28.0, 35.0] | 34.0 [31.0, 37.0] | 0.40 |
| BMI | 19.8 [18.6, 21.3] | 20.4 [18.1, 23.4] | 0.06 | 19.8 [18.4, 21.5] | 20.4 [18.1, 20.9] | 0.12 |
| Duration of SLE (days) | 2614 [1514, 4985] | 2548 [1003, 4277] | 0.03 | 2868.0 [1930.3, 4110.0] | 2343.0 [1003.0, 4214.0] | 0.19 |
| Smoking history (%) | 10 (10.5) | 2 (6.9) | 0.13 | 4 (11.8) | 1 (5.9) | 0.21 |
| previous spontaneous abortion (%) | 16 (16.8) | 8 (27.6) | 0.26 | 4 (11.8) | 5 (29.4) | 0.45 |
| previous anti-hypertensive med use (%) | 1 (1.1) | 5 (17.2) | 0.16 | 0 (0.0) | 2 (11.8) | 0.35 |
| Multiparous (%) | 34 (36.6) | 11 (37.9) | 0.03 | 13 (39.4) | 7 (41.2) | 0.04 |
| Infertility treatment (%) | 22 (23.2) | 8 (27.6) | 0.10 | 8 (23.5) | 4 (23.5) | <0.01 |
| Any flare at conception (%) | 1 (1.4) | 5 (19.2) | 0.61 | 0 (0.0) | 2 (14.3) | 0.58 |
| Zen/Doria remission at conception (%) | 46 (65.7) | 13 (50.0) | 0.32 | 21 (75.0) | 10 (71.4) | 0.08 |
| ***Organ manifestation*** |  |  |  |  |  |  |
| Joint/muscular manifestation (%) | 59 (62.1) | 20 (69.0) | 0.15 | 20 (58.8) | 13 (76.5) | 0.38 |
| Skin/mucocutaneous manifestation (%) | 70 (73.7) | 17 (58.6) | 0.32 | 27 (79.4) | 10 (58.8) | 0.46 |
| Renal manifestation (%) | 17 (17.9) | 15 (51.7) | 0.76 | 12 (35.3) | 5 (29.4) | 0.13 |
| lupus nephritis class III/IV (%) | 6 (6.3) | 6 (20.7) | 0.43 | 4 (11.8) | 3 (17.6) | 0.17 |
| Serositis (%) | 17 (17.9) | 8 (7.6) | 0.23 | 8 (23.5) | 6 (35.3) | 0.26 |
| neurological manifestation (%) | 8 (8.4) | 2 (6.9) | 0.06 | 4 (11.8) | 1 (5.9) | 0.21 |
| hematological manifestation (%) | 79 (83.2) | 21 (72.4) | 0.26 | 28 (82.4) | 13 (76.5) | 0.15 |
| ***Immunological manifestation*** |  |  |  |  |  |  |
| Anti-DNA Ab (%) | 61 (64.2) | 24 (82.8) | 0.43 | 29 (85.3) | 15 (88.2) | 0.09 |
| anti-RNP Ab (%) | 22 (36.7) | 8 (42.1) | 0.11 | 11 (40.7) | 6 (50.0) | 0.19 |
| anti-Sm Ab (%) | 28 (31.5) | 8 (29.6) | 0.04 | 14 (41.2) | 6 (35.3) | 0.12 |
| anti-Ro/SSA Ab (%) | 55 (59.1) | 21 (72.4) | 0.28 | 21 (61.8) | 14 (82.4) | 0.47 |
| anti-La/SSB Ab (%) | 13 (21.7) | 0 (0.0) | 0.74 | 8 (32.0) | 0 (0.0) | 0.97 |
| LAC (%) | 9 (9.8) | 4 (13.8) | 0.13 | 5 (15.6) | 4 (23.5) | 0.20 |
| anti-CL Ab (%) | 21 (23.1) | 6 (21.4) | 0.04 | 7 (21.2) | 4 (23.5) | 0.06 |
| anti-CLβ2GPI Ab (%) | 13 (14.0) | 2 (6.9) | 0.23 | 6 (17.6) | 2 (11.8) | 0.17 |
| low C3 (%) | 55 (59.1) | 18 (62.1) | 0.06 | 24 (70.6) | 10 (58.8) | 0.25 |
| low C4 (%) | 69 (74.2) | 22 (75.9) | 0.04 | 25 (73.5) | 14 (82.4) | 0.21 |

Ab: antibody, BMI: body mass index, CL: cardiolipin, LAC: lupus anticoagulant, SLE: systemic lupus erythematosus, SMD: standard mean difference,

**Supplementary Table S3**: Treatment regimen in the first trimester before and after propensity score matching

|  | Tacrolimus exposure (before matching) | | | Tacrolimus exposure (after matching) | | |
| --- | --- | --- | --- | --- | --- | --- |
|  | (-) | (+) | SMD | (-) | (+) | SMD |
| n | 95 | 29 |  | 34 | 17 |  |
| GC (mg/day) | 5.00 [1.00, 7.75] | 5.00 [5.00, 10.00] | 0.50 | 5.00 [4.00, 5.75] | 5.00 [4.00, 6.00] | 0.18 |
| HCQ (%) | 27 (28.4) | 20 (69.0) | 0.89 | 15 (44.1) | 8 (47.1) | 0.06 |
| Tac (%) | 0 (0.0) | 27 (93.1) | 5.2 | 0 (0.0) | 15 (88.2) | 3.87 |
| CyA (%) | 2 (2.1) | 0 (0.0) | 0.21 | 1 (2.9) | 0 (0.0) | 0.25 |
| AZA (%) | 6 (6.3) | 0 (0.0) | 0.37 | 3 (8.8) | 0 (0.0) | 0.35 |
| MMF (%) | 1 (1.1) | 0 (0.0) | 0.15 | 1 (2.9) | 0 (0.0) | 0.25 |
| MZR (%) | 0 (0.0) | 1 (3.4) | 0.27 | 0 (0.0) | 1 (5.9) | 0.35 |
| MTX (%) | 0 (0.0) | 0 (0.0) | <0.01 | 0 (0.0) | 0 (0.0) | <0.01 |
| BEL (%) | 1 (1.1) | 2 (6.9) | 0.30 | 0 (0.0) | 1 (5.9) | 0.35 |
| RTX/CY/PE/IVIg (%) | 0 (0.0) | 0 (0.0) | <0.01 | 0 (0.0) | 0 (0.0) | <0.01 |
| aspirin (%) | 32 (33.7) | 12 (41.4) | 0.16 | 15 (44.1) | 8 (47.1) | 0.06 |

AZA: azathioprine, BEL: belimumab, CY: cyclophosphamide, CyA: cyclosporine , GC: glucocorticoid, HCQ: hydroxychloroquine, IVIg: intravenous immunoglobulin, MMF: mycophenolate mofetil, MTX: methotrexate, MZR: mizoribine, NA: not available, PE: plasma exchange, PSL: prednisolone, SMD: standard mean difference, RTX: rituximab

**Supplementary Table S4**: Prevalence of adverse pregnancy outcome after propensity score matching

|  | Tacrolimus exposure PS matching | | | Logistic regression model | | |
| --- | --- | --- | --- | --- | --- | --- |
| Factor | (-) | (+) | *P* value | OR | 95% CI | *P* value |
| Number of patients | 34 | 17 |  |  |  |  |
| Overall APO (%) | 16 (47.1) | 8 (47.1) | 1.00 | 1.00 | 0.31-3.21 | 1.00 |
| Maternal APO (%) | 9 (26.5) | 5 (29.4) | 1.00 | 1.16 | 0.32-4.21 | 0.82 |
| Neonatal APO (%) | 14 (41.2) | 10 (58.8) | 0.37 | 2.04 | 0.63-6.66 | 0.24 |
| PROMISSE APO (%) | 6 (17.6) | 3 (17.6) | 1.00 | 1.00 | 0.22-4.61 | 1.00 |
| Flare during pregnancy (%) | 3 (8.8) | 4 (23.5) | 0.20 | 3.18 | 0.62-16.2 | 0.17 |
| Flare after delivery (%) | 1 (3.1) | 0 (0.0) | 1.00 | NA | NA | NA |
| Hypertensive Disorders of Pregnancy (%) | 5 (14.7) | 3 (17.6) | 1.00 | 1.24 | 0.26-5.96 | 0.79 |
| Preeclampsia (%) | 2 (5.9) | 1 (5.9) | 1.00 | 1.00 | 0.08-11.9 | 1.00 |
| Eclampsia (%) | 0 (0.0) | 0 (0.0) | NA | NA | NA | NA |
| HELLP syndrome (%) | 0 (0.0) | 0 (0.0) | NA | NA | NA | NA |
| Gestational DM (%) | 3 (8.8) | 3 (17.6) | 0.39 | 2.21 | 0.40-12.4 | 0.37 |
| Oligohydramnios (%) | 1 (3.0) | 1 (5.9) | 1.00 | 2.0 | 0.12-34.1 | 0.63 |
| Maternal death (%) | 0 (0.0) | 0 (0.0) | NA | NA | NA | NA |
| Live birth (%) | 32 (94.1) | 13 (76.5) | 0.087 | 0.20 | 0.03-1.25 | 0.09 |
| Total duration of gestation (days) | 266.5 [260.3, 271.8] | 263.0 [166.0, 267.0] | 0.14 | NA | NA | NA |
| Preterm birth (%) | 6 (18.8) | 3 (21.4) | 1.00 | 1.18 | 0.25-5.6 | 0.83 |
| Spontaneous abortion (%) | 0 (0.0) | 2 (12.5) | 0.10 | NA | NA | NA |
| Missed abortion (%) | 0 (0.0) | 0 (0.0) | NA | NA | NA | NA |
| Iatrogenic abortion (%) | 2 (5.9) | 2 ( 11.8) | 0.59 | 2.13 | 0.27-16.6 | 0.47 |
| Still birth (%) | 0 (0.0) | 0 (0.0) | NA | NA | NA | NA |
| Planned C section (%) | 6 (18.8) | 2 (15.4) | 1.00 | 1.0 | 0.14-4.53 | 0.79 |
| Emergency C section (%) | 9 (28.1) | 6 (46.2) | 0.42 | 2.19 | 0.58-8.33 | 0.25 |
| Height at birth (cm) | 47.3 [46.0, 49.0] | 46.0 [44.0, 48.0] | 0.24 | NA | NA | NA |
| Weight at birth (g) | 2731.0 [2465.5, 2951.5] | 2532.0 [2080.0, 2896.0] | 0.22 | NA | NA | NA |
| Low birth weight (%) | 10 (31.2) | 6 (46.2) | 0.55 | 1.89 | 0.50-7.07 | 0.35 |
| SGA (%) | 2 (6.2) | 2 (15.4) | 0.57 | 2.73 | 0.34-21.8 | 0.34 |
| Apgar score (1min) | 8.00 [8.00, 8.00] | 8.00 [8.00, 8.00] | 0.47 | NA | NA | NA |
| Apgar score (5min) | 9.00 [9.00, 9.00] | 9.00 [9.00, 9.00] | 0.48 | NA | NA | NA |

APO: adverse pregnancy outcome, CI: confidence interval, DM: diabetes mellitus, HELLP: hemolysis, elevated liver enzymes and low platelets, NA: not available, OR: odds ratio, PROMISSE: Predictors of Pregnancy Outcome: Biomarkers in Antiphospholipid Antibody Syndrome and Systemic Lupus Erythematosus, PS: propensity score, SGA: small for gestational age

**Supplementary Table S5:** Baseline characteristics of patient with lupus nephritis

|  | tacrolimus exposure | | |
| --- | --- | --- | --- |
| Factor | (-) | (+) | p value |
| n | 17 | 15 |  |
| ***Epidemiological findings*** | | | |
| Age at conception (yo) | 34.0 [32.0, 36.0] | 33.0 [31.0, 36.5] | 0.76 |
| BMI | 20.7 [19.1, 21.0] | 20.7 [19.1, 24.2] | 0.84 |
| Duration of SLE (days) | 3897.0 [2270.0, 5493.0] | 2869.0 [2220.0, 5406.0] | 0.75 |
| Smoking history (%) | 2 (11.8) | 1 (6.7) | 1.00 |
| previous spontaneous abortion (%) | 3 (17.6) | 4 (26.7) | 0.68 |
| previous anti-hypertensive med use (%) | 0 (0.0) | 4 (26.7) | 0.038 |
| Multiparous (%) | 10 (58.8) | 5 (33.3) | 0.18 |
| Infertility treatment (%) | 5 (29.4) | 5 (33.3) | 1.00 |
| Any flare at conception (%) | 0 (0.0) | 1 (7.1) | 1.00 |
| Zen/Doria remission at conception (%) | 7 (63.6) | 5 (35.7) | 0.24 |
| ***Organ manifestation*** | | | |
| Joint/muscular manifestation (%) | 9 (52.9) | 9 (60.0) | 0.96 |
| Skin/mucocutaneous manifestation (%) | 13 (76.5) | 10 (66.7) | 0.83 |
| Renal manifestation (%) | 17 (100.0) | 15 (100.0) | NA |
| lupus nephritis class III/IV (%) | 6 (35.3) | 6 (40.0) | 1.00 |
| Serositis (%) | 4 (23.5) | 2 (13.3) | 0.66 |
| neurological manifestation (%) | 2 (11.8) | 2 (13.3) | 1.00 |
| hematological manifestation (%) | 12 (70.6) | 12 (80.0) | 0.69 |
| ***Immunological manifestation*** | | | |
| Anti-dsDNA Ab (%) | 11 (84.6) | 3 (60.0) | 0.53 |
| anti-RNP Ab (%) | 4 (33.3) | 4 (36.4) | 1.00 |
| anti-Sm Ab (%) | 4 (23.5) | 5 (38.5) | 0.44 |
| anti-Ro/SSA Ab (%) | 5 (31.2) | 12 (80.0) | 0.011 |
| anti-La/SSB Ab (%) | 3 (30.0) | 0 (0.0) | 0.21 |
| LAC (%) | 3 (18.8) | 0 (0.0) | 0.23 |
| anti-CL Ab (%) | 3 (17.6) | 2 (14.3) | 1.00 |
| anti-CLβ2GPI Ab (%) | 2 (11.8) | 0 (0.0) | 0.90 |
| low C3 (%) | 13 (76.5) | 11 (73.3) | 1.00 |
| low C4 (%) | 11 (64.7) | 11 (73.3) | 0.71 |

BMI: body mass index, CL: cardiolipin, LAC: lupus anticoagulant, NA: not available, SLE: systemic lupus erythematosus

**Supplementary Table S6**: treatment regimen in the first trimester in patient with lupus nephritis

|  | Tacrolimus exposure | | |
| --- | --- | --- | --- |
|  | (-) | (+) | p value |
| n | 17 | 15 |  |
| GC (mg/day) (PSL equivalent) | 5.00 [3.00, 7.00] | 6.00 [5.00, 9.50] | 0.17 |
| HCQ (%) | 5 (29.4) | 11 (73.3) | 0.034 |
| Tac (%) | 0 (0.0) | 15 (100.0) | <0.01 |
| CyA (%) | 2 (11.8) | 0 (0.0) | 0.49 |
| AZA (%) | 1 (5.9) | 0 (0.0) | 1.00 |
| MMF (%) | 0 (0.0) | 0 (0.0) | NA |
| MZR (%) | 0 (0.0) | 0 (0.0) | NA |
| MTX (%) | 0 (0.0) | 0 (0.0) | NA |
| BEL (%) | 0 (0.0) | 2 (13.3) | 0.21 |
| RTX/CY/PE/IVIg (%) | 0 (0.0) | 0 (0.0) | NA |
| aspirin (%) | 7 (41.2) | 5 (33.3) | 0.93 |

AZA: azathioprine, BEL: belimumab, CY: cyclophosphamide, CyA: cyclosporine , GC: glucocorticoid, HCQ: hydroxychloroquine, IVIg: intravenous immunoglobulin, MMF: mycophenolate mofetil, MTX: methotrexate, MZR: mizoribine, NA: not available, PE: plasma exchange, PSL: prednisolone, RTX: rituximab
